# Partial Enteral Nutrition for Treating Anal Fistulas in a Boy With Crohn’s Disease Without Luminal Involvement: A Case Report

**DOI:** 10.7759/cureus.76898

**Published:** 2025-01-04

**Authors:** Yuji Fujita, Hideki Kumagai, Takeshi Yamaguchi, Kan Suzuki, Kazuyuki Ishida, Yui Akemoto, Shigemi Yoshihara

**Affiliations:** 1 Department of Pediatrics, Dokkyo Medical University, Mibu, JPN; 2 Department of Pediatrics, Jichi Medical University, Shimotsuke, JPN; 3 Division of Pediatric Surgery, Surgical Oncology Graduate School of Medicine, Dokkyo Medical University, Mibu, JPN; 4 Department of Diagnostic Pathology, Dokkyo Medical University, Mibu, JPN; 5 Department of Anatomic Pathology, Hirosaki University Graduate School of Medicine, Hirosaki, JPN; 6 Department of Pediatrics, Dokkyo Medical University Hospital, Mibu, JPN

**Keywords:** biopsy, crohn’s disease, partial enteral nutritional therapy, pediatrics, perianal fistula

## Abstract

Crohn’s disease (CD) has various clinical manifestations, including anal fistulae and perianal abscesses. While perianal abscesses typically occur during infancy, pediatricians should remain alert to the possibility of CD when perianal abscesses appear outside the usual age of onset. We report the case of a three-year-old boy with refractory anal fistulas who exhibited no gastrointestinal symptoms or growth failure at presentation. Gastrointestinal endoscopy revealed no significant mucosal abnormalities, but histopathological examination identified noncaseating epithelioid cell granulomas in the terminal ileum. Based on the presence of characteristic perianal lesions and these granulomas, the patient was diagnosed with CD. Partial enteral nutrition (PEN) therapy led to significant improvement in the anal fistulas. CD presenting solely with perianal fistulas and no luminal involvement is rare, particularly in young patients. This case underscores the importance of histopathological evaluation, including step biopsies, in patients with anal fistulas, even in the absence of gastrointestinal symptoms or endoscopic abnormalities. Additionally, it highlights the potential efficacy of PEN therapy for managing perianal fistulas without luminal involvement, as demonstrated by the patient’s marked improvement with this approach alone.

## Introduction

Crohn’s disease (CD) can present with a range of clinical manifestations, including the development of anal fistulas or perianal abscesses. Perianal abscesses typically occur during infancy and often resolve by the age of one year. However, pediatricians should remain vigilant for the possibility of CD when perianal abscesses present outside the usual age range.

Diagnosing CD as the underlying cause of anal fistulas typically requires gastrointestinal endoscopy. However, characteristic findings of CD, such as longitudinal ulcers or cobblestone patterns, may not always be evident on endoscopy [1]. In such cases, the presence of epithelioid cell granulomas on histopathological examination can aid in diagnosis. As a result, biopsies from all sites are recommended, regardless of whether gastrointestinal inflammation is observed during endoscopy [2,3].

Treatment of perianal abscesses and fistulas associated with CD often involves antibiotics, biological therapies, and surgical interventions such as seton drainage. While the efficacy of enteral nutritional therapy for perianal fistulas has not been firmly established, there are reports suggesting its potential benefits.

Here, we report the case of a pediatric patient with a refractory anal fistula that led to a diagnosis of CD despite the absence of abnormal endoscopic findings. Notably, in this case, the refractory anal fistula was successfully treated with partial enteral nutrition (PEN) therapy alone, and remission has been maintained for over 1.5 years.

## Case presentation

A three-year-old boy presented with redness, swelling, and pain in his buttocks, followed by drainage, and was referred to the pediatric surgery department of our hospital with suspected perianal fistulas, three weeks after the onset of anal symptoms. At presentation, he had neither gastrointestinal symptoms nor growth failure. Additionally, he had no family history of inflammatory bowel disease (IBD) or immunodeficiency. Physical examination revealed a reddish mass, approximately 3 cm in size, at the 3 o’clock position of the anus, and an induration of approximately 1 cm at the 9 o’clock position. The pain was more severe at the 3 o’clock position, prompting us to incise and drain the lesion. Blood examination revealed the following results: WBC count of 10,600/µL (neutrophils 44%, lymphocytes 52%), hemoglobin 13.0 g/dL, platelet count of 34.7 × 10⁴/µL, albumin 4.5 g/dL, and CRP ≤0.01 mg/dL. Stool examinations revealed occult blood at 880 ng/mL (+) and calprotectin at 65.2 mg/kg (Table 1).

**Table 1:** Laboratory data of the patient A blood examination revealed no abnormal findings. ALT: alanine aminotransferase; ANCA: antineutrophil cytoplasmic antibody; AST: aspartate aminotransferase; BUN: blood urea nitrogen; MPO: myeloperoxidase; PR3: protein 3

Hematology		Biochemistry		Immunology	
CBC		AST	24 (10-40) U/l	IgG	1,301 (870-1,700) mg/dl
WBC	10,600 (3,900-9,800)/µl	ALT	8 (5-40) U/l	MPO-ANCA	<1.0 (<3.5) U/ml
Neutrophil	44 (40-74)%	Albumin	4.5 (3.8-5.2) g/dl	PR3-ANCA	<1.0 (<3.5) U/ml
Lymphocyte	51.5 (18-59)%	BUN	11.5 (8-22) mg/dl	Neutrophil function	Normal
Hemoglobin	13 (13.5-17.6) g/dl	Creatinine	0.32 (0.61-1.04) mg/dl	Stool test	
Platelet	34.7 (13.1-36.2) × 10⁴/µl	CRP	<0.01 (<0.14) mg/dl	Fecal calprotectin	65.2 (<50) mg/kg

**Figure 1:**
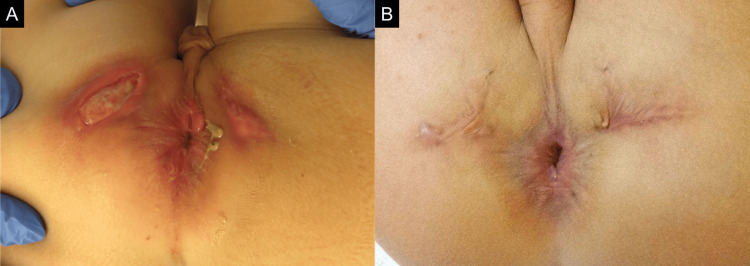
Results of physical examination (A) The patient presented with a small ulcer at the 3 o’clock position and a large ulcer at the 9 o’clock position of the anus. (B) PEN therapy led to an improvement in clinical symptoms. PEN: partial enteral nutrition

Although a Chinese herbal medicine (Hainosankyuto, TJ-122; Tsumura & Co, Tokyo, Japan) was applied [4,5], there was no improvement in the anal fistula. A large ulcer was noted at the 9 o’clock position (Figure 1A).

**Figure 2:**
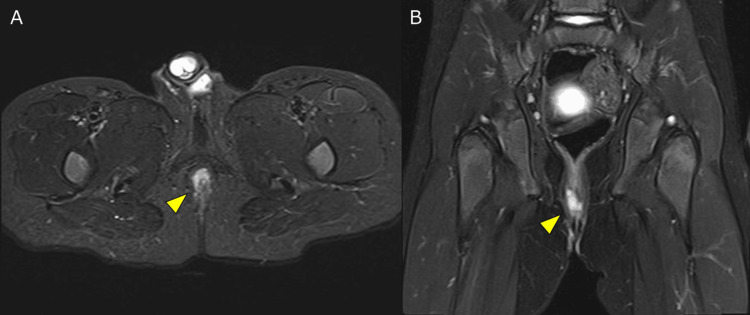
Results of MRI Short T1 inversion recovery MRI of the buttocks showing complex anal fistulas and a perianal abscess (yellow arrowhead): (A) Sagittal view. (B) Coronal view.

An MRI of the buttocks subsequently revealed a complex anal fistula and a perianal abscess (Figure 2).

**Figure 3:**
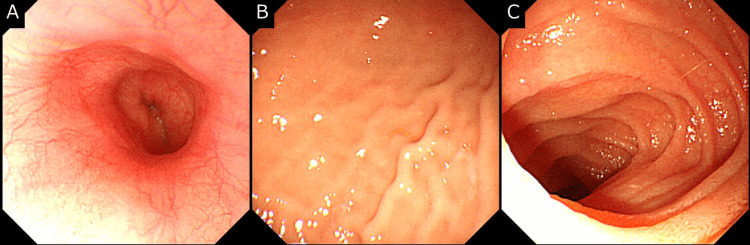
Esophagogastroduodenoscopy results Esophagogastroduodenoscopy of (A) the esophagus, (B) the stomach, and (C) the duodenum revealed no abnormal findings.

**Figure 4:**
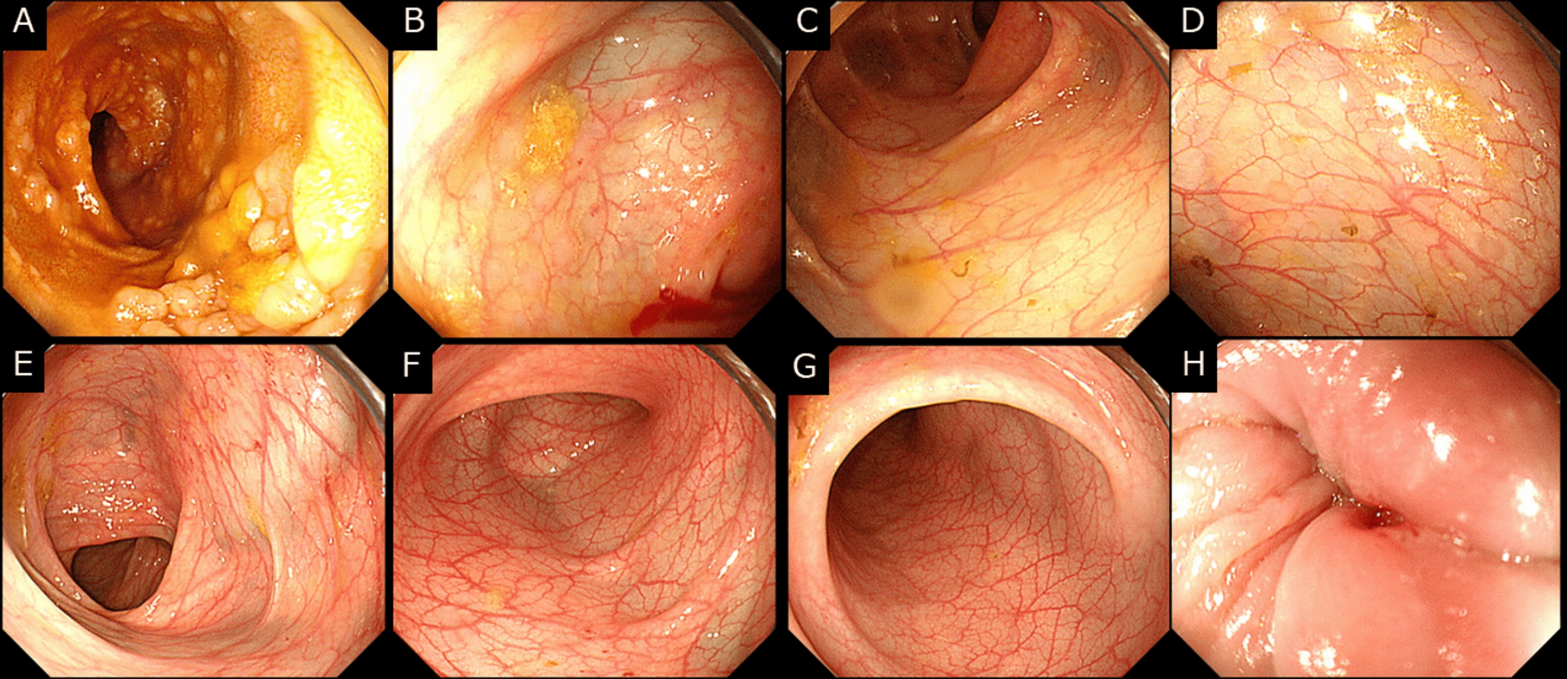
Colonoscopy results Colonoscopy revealed no abnormal findings in (A) the terminal ileum, (B) the cecum, (C) the ascending colon, (D) the transverse colon, (E) the descending colon, (F) the sigmoid colon, (G) the rectum, or (H) the anus.

Considering the possibility of CD, endoscopic examinations were performed four months after the onset of his anal symptoms. Esophagogastroduodenoscopy and colonoscopy revealed no abnormalities (Figure 3, Figure 4).

**Figure 5:**
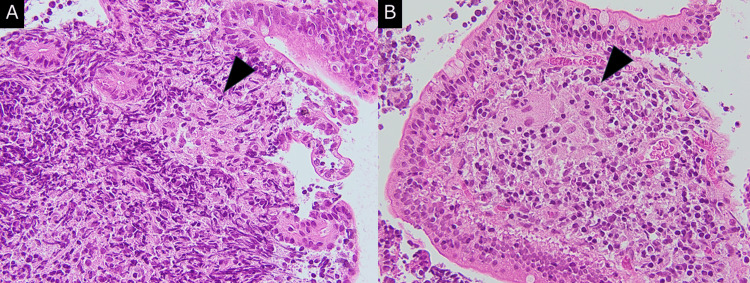
Histopathological examination results Histopathological examination revealed noncaseating epithelioid cell granulomas (arrowhead) in the terminal ileum. Original magnification: ×400.

Histopathological examination revealed noncaseating epithelioid cell granulomas in the terminal ileum (Figure 5).

Genetic analysis was performed because very early onset IBD, which may be associated with a monogenic abnormality, is defined as onset before the age of six years. Genetic analysis of genes covered by Japanese medical insurance (IL10, IL10RA, IL10RB, NFAT5, TGFB1, RIPK1, FOXP3, IL2RA, CTLA4, LRBA, WAS, XIAP, CYBA, CYBB, NCF2, NCF4, and TNFAIP3) associated with very early onset IBD did not reveal any pathogenic mutations. The patient was diagnosed with CD based on characteristic perianal lesions and noncaseating epithelioid cell granulomas [2].

PEN therapy, including an elemental diet of 300 kcal/day and a fat-restricted diet, improved the clinical symptoms of anal fistulas (Figure 1B). Subsequent retrograde double-balloon enteroscopy revealed several small erosions in the ileum, but no lesions suggestive of CD. A perianal abscess recurred transiently when the patient was infected with adenovirus enteritis, but it improved rapidly with PEN alone. MRI thereafter revealed that the abscess had disappeared, and the patient maintained clinical remission with PEN, without requiring any additional treatments such as antibiotics, drainage, or seton placement, for more than two years. Written informed consent for the publication of this case report was obtained from the patient’s parents.

## Discussion

The clinical course of the present case highlights two key points: first, histopathological evaluation using step biopsy can lead to a diagnosis of CD even in the absence of gastrointestinal symptoms and endoscopic findings; and second, PEN therapy is effective for treating anal fistulas, even when there is no luminal involvement.

Van Limbergen et al. [6] studied the phenotype at the time of diagnosis in 273 pediatric CD cases and found that seven (2.6%) were oral type, four (1.5%) were oral + perianal type, three (1.1%) were perianal type, and 259 (94.8%) had luminal involvement. This study also noted that oral and/or perianal types were significantly more common in children under eight years of age (13.2%) compared to those over eight years (2.7%). Pediatricians should be aware that perianal-type CD can present as refractory anal fistulas, even in the absence of gastrointestinal symptoms like diarrhea or bloody stools.

The European Society for Paediatric Gastroenterology Hepatology and Nutrition (ESPGHAN) recommends obtaining biopsies from all sites, regardless of whether gastrointestinal inflammation is visible on endoscopy [2]. This guideline also suggests that a peripheral phenotype with epithelioid cell granulomas may represent asymptomatic or rapidly progressive luminal CD. This case underscores the importance of histopathological evaluation using step biopsy, even without gastrointestinal symptoms or endoscopic abnormalities.

Nutritional therapy, including elemental dietary supplements and lipid-restricted diets, is a fundamental treatment for CD, particularly for luminal involvement. Drug therapies such as glucocorticoids, immunosuppressants, and biological agents are also key, with biological agents like infliximab being particularly important for managing perianal fistulas. Several studies have reported the effectiveness of nutritional therapy for anal fistulas. In terms of short-term effectiveness, exclusive enteral nutrition (EEN) improved perianal fistulas in four of six adult cases, with one case showing complete resolution [7]. For long-term effectiveness, perianal fistulas improved in nine of 12 adult patients with EEN, although recurrence occurred in eight of nine patients after returning to a normal diet [8]. In pediatric cases, EEN improved anal fistulas in three children, with remission maintained even with PEN, avoiding the need for biological agents or surgery [9]. These studies suggest that changes in the intestinal microbiota may play a role in the mechanism by which nutritional therapy benefits anal fistulas, although the exact mechanism remains unclear.

Although our patient experienced a temporary relapse of the perianal abscess due to adenovirus enteritis, clinical remission has been successfully maintained for over two years with PEN therapy alone.

## Conclusions

Pediatricians and pediatric surgeons should consider CD in the differential diagnosis of perianal fistulas. This case highlights the critical role of histopathological evaluation using step biopsy in diagnosing anal fistulas, even in the absence of gastrointestinal symptoms or endoscopic abnormalities. Additionally, it suggests that PEN therapy may be effective in treating anal fistulas, even without luminal involvement, an approach that is often overlooked in such cases. Given that many CD patients with perianal fistulas are refractory to drug therapies, including biological agents, further investigation into the effectiveness of nutritional therapy for perianal fistulas is warranted.
